# An ischemia-homing bioengineered nano-scavenger for specifically alleviating multiple pathogeneses in ischemic stroke

**DOI:** 10.1186/s12951-022-01602-7

**Published:** 2022-08-31

**Authors:** Ranran Duan, Ke Sun, Fang Fang, Ning Wang, Ruya He, Yang Gao, Lijun Jing, Yanfei Li, Zhe Gong, Yaobing Yao, Tingting Luan, Chaopeng Zhang, Jinwei Zhang, Yi Zhao, Haojie Xie, Yongyan Zhou, Junfang Teng, Jinfeng Zhang, Yanjie Jia

**Affiliations:** 1 Department of Neurology, The First Affiliated Hospital of Zhengzhou University, Zhengzhou, 450052 Henan China; 2 Department of Urinary Surgery, The First Affiliated Hospital of Zhengzhou University, Zhengzhou, 450052 Henan China; 3 Key Laboratory of Molecular Medicine and Biotherapy, School of Life Sciences, Beijing Institute of Technology, Beijing, 100811 China; 4 Department of Biochemistry, College of Life Sciences, Shaanxi Normal University, Xi’an, 710062 Shanxi China; 5 The International Medical Center, The First Affiliated Hospital, Zhengzhou University, Zhengzhou, 450052 Henan China

**Keywords:** Ischemic stroke, Nano-scavenger, Microglia polarization, Reactive oxygen species elimination, Iron chelation, Neuroprotection

## Abstract

**Background:**

Ischemic stroke is one of the most serious global public health problems. However, the performance of current therapeutic regimens is limited due to their poor target specificity, narrow therapeutic time window, and compromised therapeutic effect. To overcome these barriers, we designed an ischemia-homing bioengineered nano-scavenger by camouflaging a catalase (CAT)-loaded self-assembled tannic acid (TA) nanoparticle with a M2-type microglia membrane (TPC@M2 NPs) for ischemic stroke treatment.

**Results:**

The TPC@M2 NPs can on-demand release TA molecules to chelate excessive Fe^2+^, while acid-responsively liberating CAT to synergistically scavenge multiple ROS (·OH, ·O_2_^−^, and H_2_O_2_). Besides, the M2 microglia membrane not only can be served as bioinspired therapeutic agents to repolarize M1 microglia into M2 phenotype but also endows the nano-scavenger with ischemia-homing and BBB-crossing capabilities.

**Conclusions:**

The nano-scavenger for specific clearance of multiple pathogenic elements to alleviate inflammation and protect neurons holds great promise for combating ischemic stroke and other inflammation-related diseases.

**Supplementary Information:**

The online version contains supplementary material available at 10.1186/s12951-022-01602-7.

## Background

Ischemic stroke is one of the most serious public health problems leading to high mortality and serious long-term acquired disability worldwide [1–7]. It is generally attributed to transient or permanent occlusion of the cerebral artery which would induce ischemia/reperfusion injury and further lead to severe brain damage and loss of neural functions [8, 9]. To date, the most efficient clinical treatments for ischemic stroke mainly include surgical recanalization and thrombolytic pharmacotherapy. However, the surgical intervention is often limited by high expense, invasive bleeding complications, and narrow surgical time window within 12–24 h of symptom onset [4]. Meanwhile, recombinant tissue plasminogen activator (rtPA), currently the only Food and Drug Administration (FDA)-approved effective thrombolytic drug for the management of ischemic stroke, suffers from a short half-life, poor affinity towards thrombus as well as narrow therapeutic time window within only 3–4.5 h after stroke onset [10–12]. Stem cell transplantation is a widely-used treatment approach for stroke, and it has some limitations in terms of stem cell source, cell allogeneic rejection, risk of cell tumorigenesis, cell survival and enrichment rate after cell transplantation, and choice of cell administration methods [13–15]. Compared to stem cell therapy, exosome therapy is safer, has no ethical issues, and has high biodistribution in the brain after transplantation. Although stem cell therapy and exosome therapy have achieved promising results in stroke animal models, large-scale clinical trials with long-term large samples are lacking [16, 17]. Therefore, developing effective strategies for targeted treatment of ischemic stroke with desirable therapeutic performances and low sides effects is urgently desirable but challenging.

During the past decades, tremendous progress has been made to understand the different mechanisms associated with the pathogenesis of ischemic stroke. Microglia cells are the resident immune cells in the brain which are imperative regulators of central nervous system (CNS) homeostasis, existing in two polarized phenotypes including a pro-inflammatory M1 phenotype and an anti-inflammatory M2 phenotype [18–24]. Particularly, over-activated M1 phenotype microglia cells play an important role in ischemic stroke injury which can trigger neuroinflammation and neuronal dysfunction [23, 25–29]. On the other hand, the reperfusion initiates overproduction of reactive oxygen species (ROS) in the ischemic site including hydrogen peroxide (H_2_O_2_), superoxide anion (·O_2_^−^), and hydroxyl radical (·OH), which is another well-known crucial pathological cause of ischemic stroke [30–33]. The upregulated ROS can not only directly induce mitochondrial damage and neuron apoptosis but also serve as important signaling regulators to stimulate the activation of resident microglia and infiltrated peripheral leukocytes via the nuclear factor-κB (NF-κB) signaling pathway, thus ultimately aggravating the inflammation [20, 34–39]. Furthermore, excessive ferrous ions (Fe^2+^) in microglia cells caused by hemolysis could continuously catalyze intracellular H_2_O_2_ into ·OH and ·O_2_^−^ via Fenton reactions, which is also responsible for lasting neurological impairment and ischemic injury [40]. Collectively, these different pathogenic mechanisms offer new opportunities for the treatment of ischemic stroke.

Recently, a variety of different advanced nanomaterials including ceria, iron oxide-based, Prussian blue, and polymer nanoparticles have been designed for the treatment of ischemic stroke due to their favorable physicochemical and physiological properties [28, 41–49]. Although attractive, there are still some crucial limitations such as poor targeting of ischemic areas, low permeability to the blood–brain barrier (BBB), and potential neurotoxicity, making their wide clinical translation less feasible [50, 51]. More importantly, most existing nanomaterials for stroke treatment merely rely on monotherapy with a single mechanism where only removing ROS is commonly used, which would further compromise their practical therapeutic efficiency [52–54]. Thus, we aim to exploit a highly efficient nano-scavenger equipping with both targeting capacity and multiple therapeutic mechanisms against ischemic stroke, which has never been reported yet to the best of our knowledge.

**Scheme 1:**
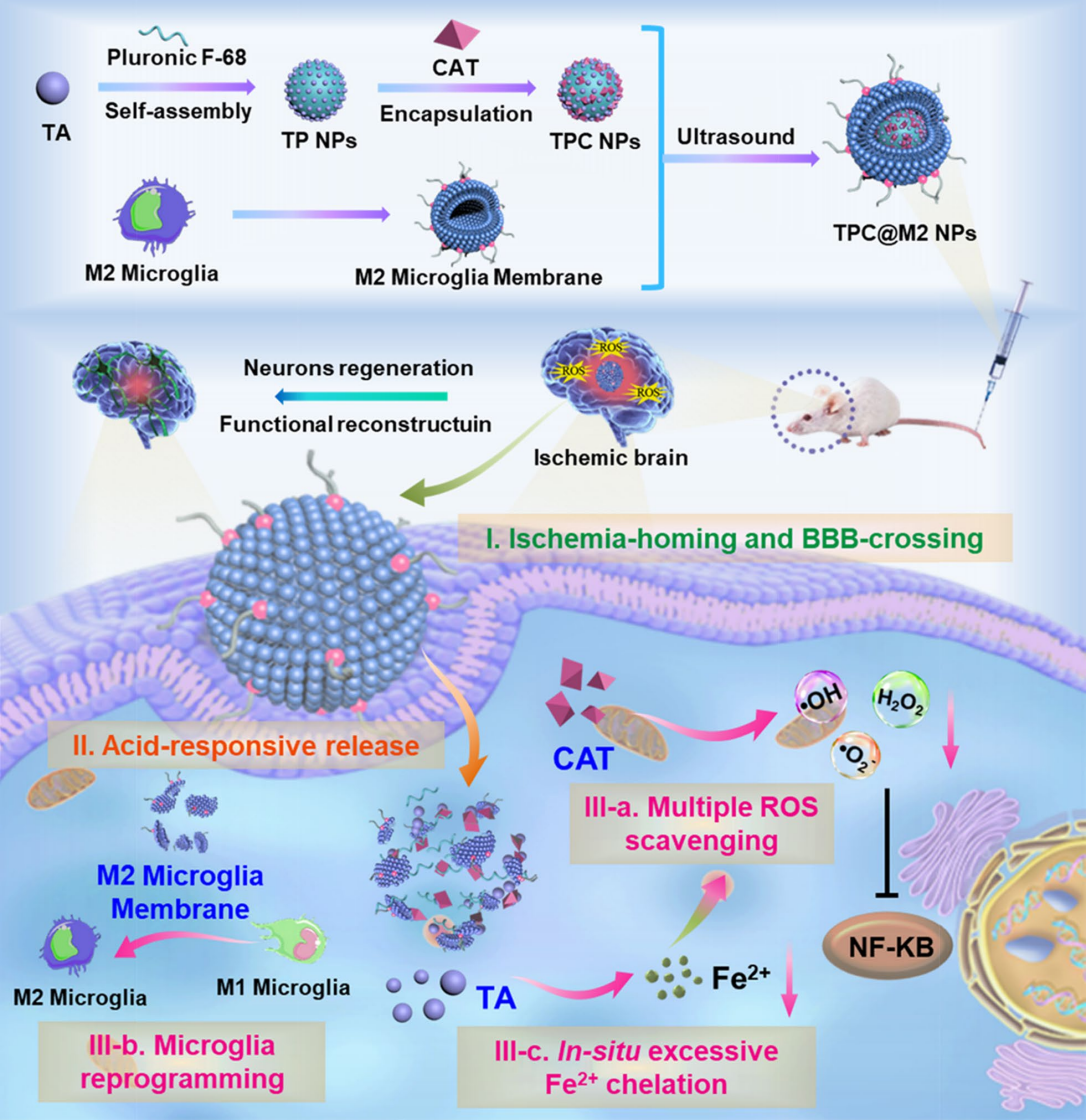
Illustration of the ischemia-homing bioengineered nano-scavenger (TPC@M2 NPs) preparation and its neuroprotection effect via specific clearance of multiple pathogenic elements including overproduced multiple-ROS, excessive Fe^2+^ and highly expressed inflammatory microglia against ischemic stroke

In this work, we proposed a bioengineered nano-scavenger for specific clearance of multiple pathogenic elements including overproduced multiple-ROS, excessive Fe^2+^, and inflammatory microglia for targeted ischemic stroke treatment (Scheme 1). First, the nano-scavenger was prepared by camouflaging a self-assembled nanoparticle core with a M2-type microglia membrane (termed as TPC@M2 NPs) which shows intrinsic ischemia-homing and BBB-crossing capabilities. The nano-scavenger core was self-assembled from a natural plant polyphenol tannic acid (TA) with a poloxamer 188 (Pluronic F-68, intravenous excipients) surfactant to further encapsulate catalase (CAT) (termed as TP NPs and TPC NPs respectively), which exhibits acid-responsive properties for on-demand release of the loaded molecules. TA, which is recognized as Generally Recognized as Safe (GRAS) by the U.S. FDA, has strong free radical scavenging and antioxidant abilities. Moreover, TA is rich in catechol and polytriphenol groups, can strongly chelate different metal ions via coordination bonds and may swiftly form a stable five-element ring complex with metal iron ions. As such, the ROS scavenging and iron ion complexation properties of TA could actively modulate the ROS boosting and iron homeostasis in the ischemic site. While the liberated CAT can combine with the decreased Fe^2+^ to clear the over-produced multiple pathogenic ROS (·OH, ·O_2_^−^, and H_2_O_2_) [55, 56]. Notably, the M2 microglia membranes can be also served as a bioinspired therapeutic agent by secreting anti-inflammatory cytokines as well as repolarizing M1 microglia into M2 phenotype [57]. Taken together, the resulting nano-scavenger can synergistically alleviate inflammation and protect neurons by integrating multiple therapeutic mechanisms, which could be a promising strategy for ischemic stroke treatment.

## Results and discussion

### Preparation, characterization, and acid-responsive multiple ROS/Fe^2+^-clearing capabilities of the TPC@M2 NPs

**Fig. 1:**
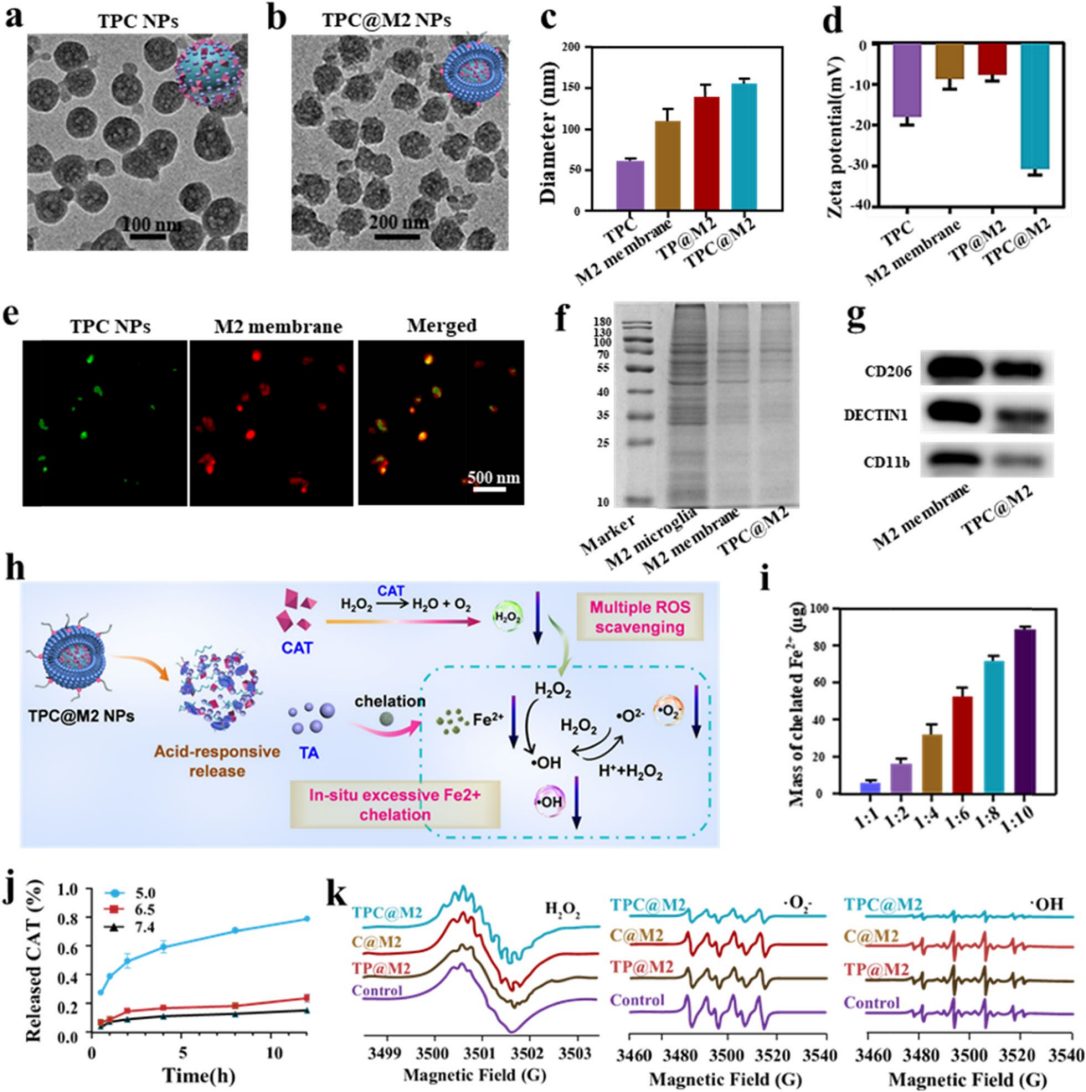
Characterizations of the TPC@M2 NPs. The TEM images of **a** TPC NPs and **b** TPC@M2 NPs. **c** The sizes and **d** corresponding zeta potentials of different samples (n = 3). **e** CLSM images of the co-localization of TPC NPs core (green) and M2 microglia membrane (red) in TPC@M2 NPs. **f** SDS-PAGE electrophoresis analysis of proteins in M2 microglia, M2 membrane, and TPC@M2 NPs. **g** Western blotting for M2 microglia-specific membrane proteins in TPC@M2 NPs. **h** The multiple-ROS/Fe^2+^ scavenging mechanism of the TPC@M2 NPs. **i** The content of Fe^2+^ chelated by the TPC NPs at different molar ratio of TPC/Fe^2+^ (n = 3). **j** Release of CAT from TPC@M2 NPs after incubation in different pH of PBS for 12 h, respectively (n = 5). **k** Multiple ROS scavenging properties of different groups. Results are presented as means ± s.d. *P < 0.05, **P < 0.01, and ***P < 0.001 determined by Student’s t-test

The preparation process and composition of the TPC@M2 NPs are shown in Scheme 1. The transmission electron microscopy (TEM) results showed that the TP NPs self-assembled from the TA molecule with Pluronic F-68 displayed uniform spherical structures with a size of ~ 60 nm (Additional file 1: Fig. S1). Subsequently, the CAT as a ROS-scavenger was inserted into the TP NPs (termed as TPC NPs), exhibiting no obvious shape change (Fig. 1a). Bicinchoninic acid protein assay was used to estimate encapsulation efficiency and loading capability of CAT, which were 30% and 285 μg enzymes per milligram of TA, respectively.

To further endow the TPC NPs with ischemia-homing and microglia reprogramming abilities, the M2 microglia membrane was extracted and coated on the surface of the TPC NPs, ultimately obtained the bioengineered nano-scavenger (termed as TPC@M2 NPs). The morphology and zeta potential of TPC@M2 NPs were studied by TEM and dynamic light scattering (DLS), which indicated that the size of the TPC@M2 NPs (~ 160 nm) was larger than that of the uncoated TPC NPs (Fig. 1b, c, and Additional file 1: Fig. S2), while the zeta potential of TPC@M2 NPs decreased to − 30 mV (Fig. 1d). TP NPs coated with M2 microglia membrane (termed as TP@M2 NPs) and M2 membrane were used as control (Fig. 1d). To estimate the successful modification of the M2 microglia membrane, the TPC NPs were labeled with FITC and the M2 microglia membrane was labeled with Dil dye. The co-localization of FITC and Dil fluorescence signals could be observed by the confocal laser scanning microscopy (CLSM), confirming the TPC NPs were ultimately coated with the M2 microglia membrane (Fig. 1e). In addition, the SDS-PAGE analysis also indicated that protein bands in the TPC@M2 NPs were nearly identical to that of the pure M2 microglia membrane (Fig. 1f). For further confirmation, we investigated the M2 microglia-specific membrane proteins (CD206, Dectin1, CD11b) by Western blot. As shown in Fig. 1g, all the three proteins were detected in the TPC@M2 NPs, further suggesting that the TPC@M2 NPs were successfully enveloped by the M2 microglia membrane. Furthermore, the TPC@M2 NPs displayed great biological stability (Additional file 1: Fig. S3) and good biocompatibility (Additional file 1: Fig. S4).

After confirming the successful preparation of the TPC@M2 NPs, their multiple-ROS/Fe^2+^ scavenging potency was investigated and the detailed mechanism was displayed in Fig. 1h. First, we investigated the Fe^2+^ chelation ability of the TPC NPs. It was found that the Fe^2+^ content chelated by the TPC NPs increased significantly with the enhancement of the NPs/Fe^2+^ ratio (Fig. 1i), which indicated that the TPC NPs showed strong chelation ability to Fe^2+^. We then measured the CAT release from the TPC@M2 NPs under different pH conditions. As depicted in Fig. 1j, the release of CAT was greatly improved in pH 5.0 than that of pH 7.4 and pH 6.5, owing to the acid-responsive collapse of the TPC@M2 NPs in pH 5.0. As determined by the Góth method, the TPC@M2 maintained about 32% of enzymatic activity of equivalent free CAT molecules after being encapsulated into the NPs. While a rapid rise of O_2_ concentration was observed after the addition of the TPC@M2 NPs into the H_2_O_2_ aqueous solution, demonstrating that the encapsulated CAT still possessed catalytic activity toward H_2_O_2_ with a concentration-dependent manner (Additional file 1: Fig. S5). We further evaluated the enzymatic stability of the TPC@M2 NPs compared to free CAT against proteases. CAT encapsulated in the TPC@M2 NPs was well protected against protease K and kept ≈ 62% of its initial activity after incubation with protease K for 3 h, while free CAT was completely lost activity after the same treatment (Additional file 1: Fig. S6). Therefore, although the relative activity of CAT decreased after being encapsulated into the TPC@M2 NPs, its enzymatic stability was greatly enhanced. Then, the multiple ROS (·OH, ·O_2_^−^, and H_2_O_2_) scavenging abilities of the as-fabricated NPs was confirmed by ESR results (Fig. 1k). Overall, these results demonstrated that the as-prepared TPC@M2 NPs can not only efficiently chelate Fe^2+^ but also robustly scavenge multiple ROS, which further provides the possibility for phenotype transformation of microglia cells.

### The TPC@M2 NPs scavenge multiple ROS and chelate Fe^2+^ in the microglial cells

**Fig. 2:**
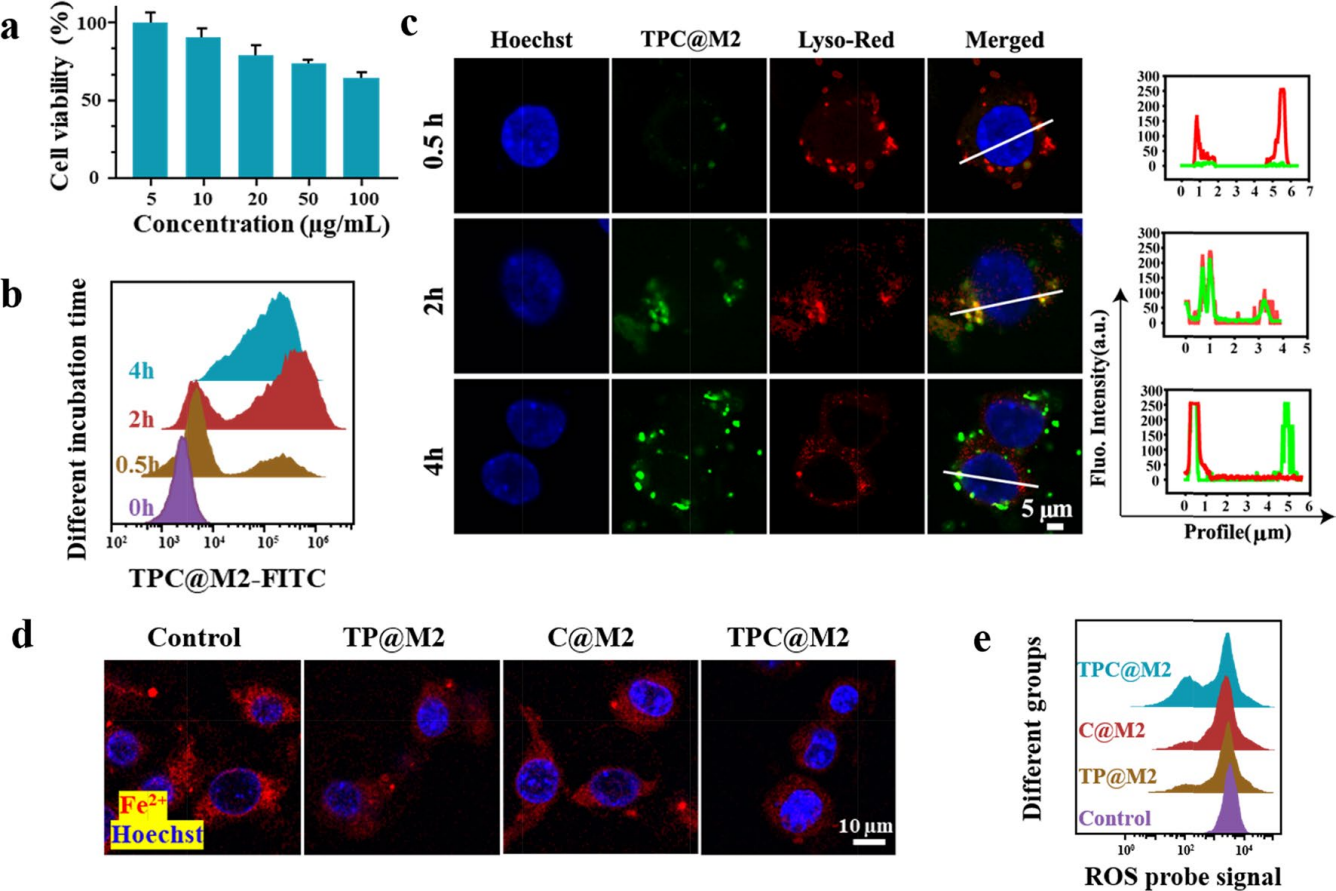
Multiple ROS scavenging and Fe^2+^-chelating capacities of the TPC@M2 NPs in the microglial cells. **a** The cell viabilities of BV-2 cells treated with different concentrations of the TPC@M2 NPs for 24 h (n = 6). **b** Flow cytometry analysis of the BV-2 cells incubated with the TPC@M2 NPs for 0 h, 0.5 h, 2 h, 4 h. **c** CLSM images of cellular distribution of the TPC@M2 NPs in the M1-type BV-2 cells and the corresponding co-localization of TPC@M2 NPs and the lysosome. Scale bar: 5 μm. **d** CLSM image of Fe^2+^ levels in the M1-type BV-2 cells after different treatments. Scale bar: 25 μm. **e** Flow cytometry analysis of ROS levels in the M1-type BV-2 cells after different treatments. Results are presented as means ± s.d. *P < 0.05, **P < 0.01, and ***P < 0.001 determined by Student’s t-test

To confirm the intracellular multiple ROS scavenging and Fe^2+^ chelating capacities of the TPC@M2 NPs, we selected the murine microglial cell lines (BV-2 cells) for the following in vitro experiments. To establish the ROS and Fe^2+^ overexpressed M1-type cell model, BV-2 cells were simultaneously pre-treated with FeCl_2_ and H_2_O_2_. After incubation with the TPC@M2 NPs in different concentrations for 24 h, cytotoxicity of the pre-treated BV-2 cells was detected. As presented in Fig. 2a, the TPC@M2 NPs showed low cytotoxicity on BV-2 cells. Moreover, the TPC@M2 NPs displayed time-dependent endocytosis (Fig. 2b) and lysosomal release behavior (Fig. 2c). According to CLSM results, the co-localization rate of the TPC@M2 NPs and the lysosome was highest at 2 h (about 80%) while decreased at 4 h (about 25%). These results revealed that the TPC@M2 NPs could achieve acid-responsive release of the different components within the lysosome. Afterward, to further characterize the high multiple ROS/Fe^2+^ scavenging ability of the TPC@M2 NPs in activated BV-2 cells. Cellular Fe^2+^ and ROS levels of the as-fabricated bioengineered NPs-treated M1-type BV-2 cells were respectively determined. Intriguingly, the content of Fe^2+^ (Fig. 2d and Additional file 1: Fig. S7) and ROS (Fig. 2e) in M1-type BV-2 cells treated with the TPC@M2 NPs were lower than that of other groups, indicating that the released TA and CAT in the acidic environment of lysosomes played an important role in Fe^2+^ chelation and multiple ROS scavenging.

### Reprogramming the microglial cell phenotype from M1 to M2 for neuroprotection

**Fig. 3:**
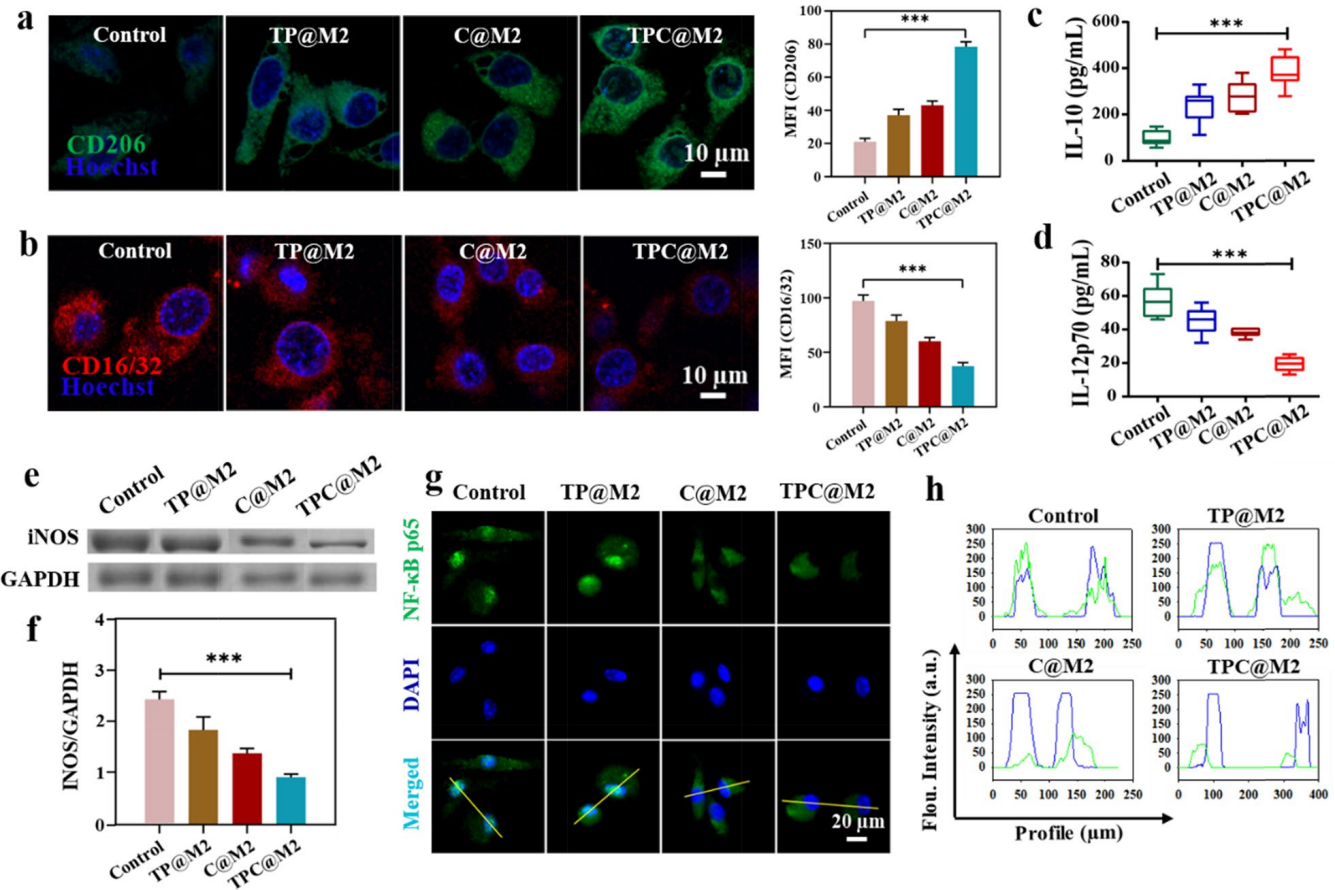
Reprogramming the microglial cell phenotype from M1 to M2-type by the TPC@M2 NPs. CLSM images and the corresponding mean fluorescence intensity (MFI) of **a** the M2 biomarkers (CD206^+^: green) and **b** the M1 biomarkers (CD16/32^+^: red) on the BV-2 cells treated with different groups. Relative expression levels of **c** the anti-inflammatory cytokine IL-10 (**d**) and the pro-inflammatory cytokine IL-12p70 in the BV-2 cells treated with different groups (n = 4). **e** SDS-PAGE protein analysis and **f** gray value semi-quantitative of iNOS expression in BV-2 cells after treatment with different groups for 48 h (n = 3). **g** CLSM images of the distribution of NF-κB p65 in the BV-2 cells after different treatments and **h** the corresponding co-localization of NF-κB and nucleus. Results are presented as means ± s.d. *P < 0.05, **P < 0.01, and ***P < 0.001 determined by Student’s t-test

To further investigate the phenotypic transformation effect of the TPC@M2 NPs on the microglial cells, CLSM studies showed that the TPC@M2 NPs could induce the increase of M2-type BV-2 cells (CD206^+^) and the decrease of M1-type BV-2 cells (CD16/32^+^) (Fig. 3a, b), which was consistent with the flow cytometry results (Additional file 1: Fig. S8). Meanwhile, the secretion of anti-inflammatory cytokine interleukin-10 (IL-10) is accordingly increased (Fig. 3c) while expression of the pro-inflammatory cytokine interleukin 12p70 (IL-12p70) is decreased in activated BV-2 cells (Fig. 3d). Phenotypic transformation of microglia cells was also confirmed by the lowest expression of inducible nitric oxide synthase (iNOS) in the TPC@M2 NPs treated group (Fig. 3e, f). Considering the down-regulation of pro-inflammatory genes, we speculated that the TPC@M2 NPs regulate microglia cells phenotypic transformation by blocking ROS-triggered inflammatory signaling pathways. It has been well-studied that the NF-κB is an important inflammatory regulator, which can be activated by ROS to induce a series of pro-inflammatory activities. Therefore, we investigated the nuclear transfer of NF-κB p65 in microglia after different NPs treatments. According to CLSM images and the spatial co-localization of NF-κB p65 and nucleus (Fig. 3g, h), nuclear transfer of NF-κB p65 was blocked in the TPC@M2 and C@M2 groups, while not in the TP@M2 treatment group, indicating that the CAT-encapsulated samples (the TPC@M2 and C@M2 NPs) directly blocked the activation of NF-κB p65 by eliminating ROS, thus inhibiting the anti-inflammatory phenotype of microglia cell.

### BBB-crossing and ischemia-homing capabilities of the TPC@M2 NPs

**Fig. 4:**
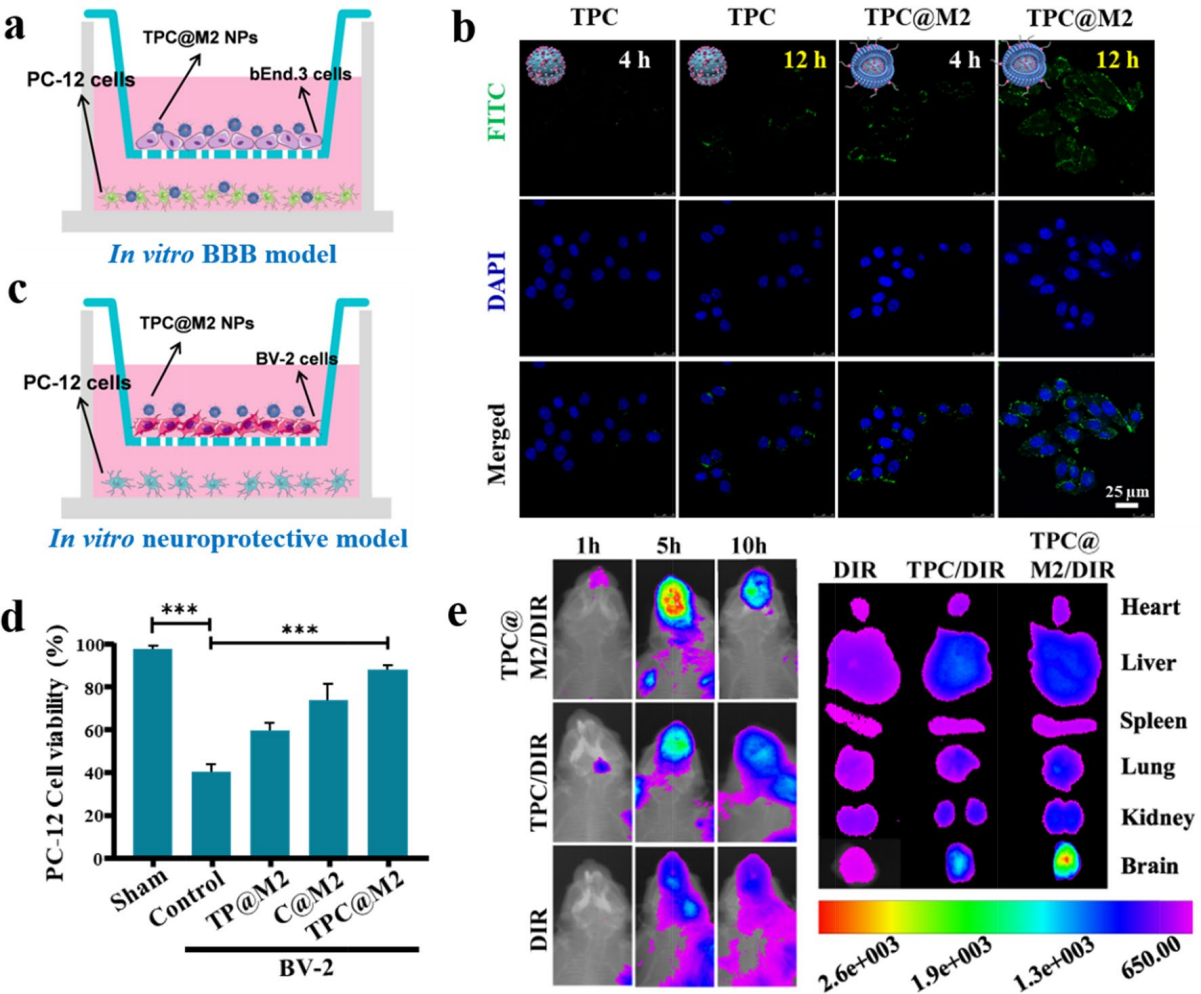
BBB-crossing and ischemia-homing capabilities of the TPC@M2 NPs. **a** Schematic of the transwell assay for constructing the BBB model in vitro. **b** CLSM images of the PC-12 cells treated with different treatments, scale bar: 25 μm. **c** Diagram of BV-2 cells and PC-12 cells co-cultured in a transwell dish. **d** CCK-8 assay of PC-12 cells after cocultured with BV-2 cells which pre-treated with the TPC@M2 NPs (n = 6). **e** Optical imaging of MCAO rats and the main tissues after injection of DIR/ TPC/DIR and TPC@M2/DIR (with the same dosage of DIR) (n = 3). Results are presented as means ± s.d. *P < 0.05, **P < 0.01, and ***P < 0.001 determined by Student’s t-test

The biosafety of nanoparticles has always been a concern, so we extracted rat primary nerve cells, and the cytotoxicity of TPC@M2 NPs on primary nerve cells and rat pheochromocytoma cell lines (PC-12 cells), which is one of the most commonly used cell lines in neuroscience research, was investigated. As shown in the Additional file 1: Fig. S9, TPC@M2 NPs showed low cytotoxicity on rat primary neuronal cells and PC-12 cells. In order to avoid sacrificing more rats, PC-12 cells were used to replace rat primary neuronal cells in subsequent experiments. The high BBB-crossing and ischemia-homing capabilities of the TPC@M2 NPs are prerequisites for efficient ischemic stroke neuroprotection. Firstly, the in vitro BBB model was constructed by a polycarbonate 24-well Transwell membrane of 0.4 μm pore size, in which bEnd.3 cells were seeded at a density of 5 × 10^4^ cells/well and cultured for 3 days. An epithelial volt ohm meter was used to measure the transendothelial electrical resistance of cell monolayers, and those above 220 Ω/cm^2^ were selected for experiments. Afterwards the basolateral bottom of the transwell plate was seeded with PC-12 cells. Finally, the BBB crossing ability of TPC@M2 NPs were evaluated by CLSM in vitro (Fig. 4a). Compared to the naked TPC NPs, we observed that TPC@M2 NPs have a relatively high penetration effect across the BBB (Fig. 4b and Additional file 1: Fig. S10). Since anti-inflammatory M2-phenotype microglia cells play a neuroprotective role in stroke, we subsequently selected PC-12 cells to investigate the neuroprotective effect of microglia cell in vitro. Firstly, M1-type BV-2 cells in the upper chamber of the transwell dish was incubated with different treatment for 6 h, then a fresh medium was added for further culture for another 24 h. Meanwhile, PC-12 cells were spread in the lower chamber and co-incubated with BV-2 cells for 24 h. As elucidated in Fig. 4d, M1-type BV-2 cells treated with the TPC@M2 NPs increased the survival rate of PC-12 cells by 40% compared with the untreated group, which will be beneficial for neuroprotection and functional recovery after ischemic stroke.

Then, we used near-infrared fluorescence imaging to investigate the distribution behavior and ischemia-homing ability of the TPC@M2 NPs in middle cerebral artery occlusion (MCAO) rats, where TPC NPs and free DIR is used as a control. As shown in Fig. 4e, the fluorescence signals in the brain of the free DIR-treated mice were relatively weak. By comparison, TPC NPs treated mice showed enhanced DIR signal, while the TPC@M2 NPS treated group showed the strongest DIR signal, demonstrating the inherent inflammatory affinity and ischemia-site targeting ability of the M2 microglial cell membrane. In addition, brain accumulation of the TPC@M2 NPs was time-dependent and reached its maximum value after administration for 5 h. Fluorescence imaging of other main organs and brains of rats after 10 h showed the same trend.

### The in vivo therapeutic effects and biosafety evaluation of the TPC@M2 NPs against ischemic stroke

**Fig. 5:**
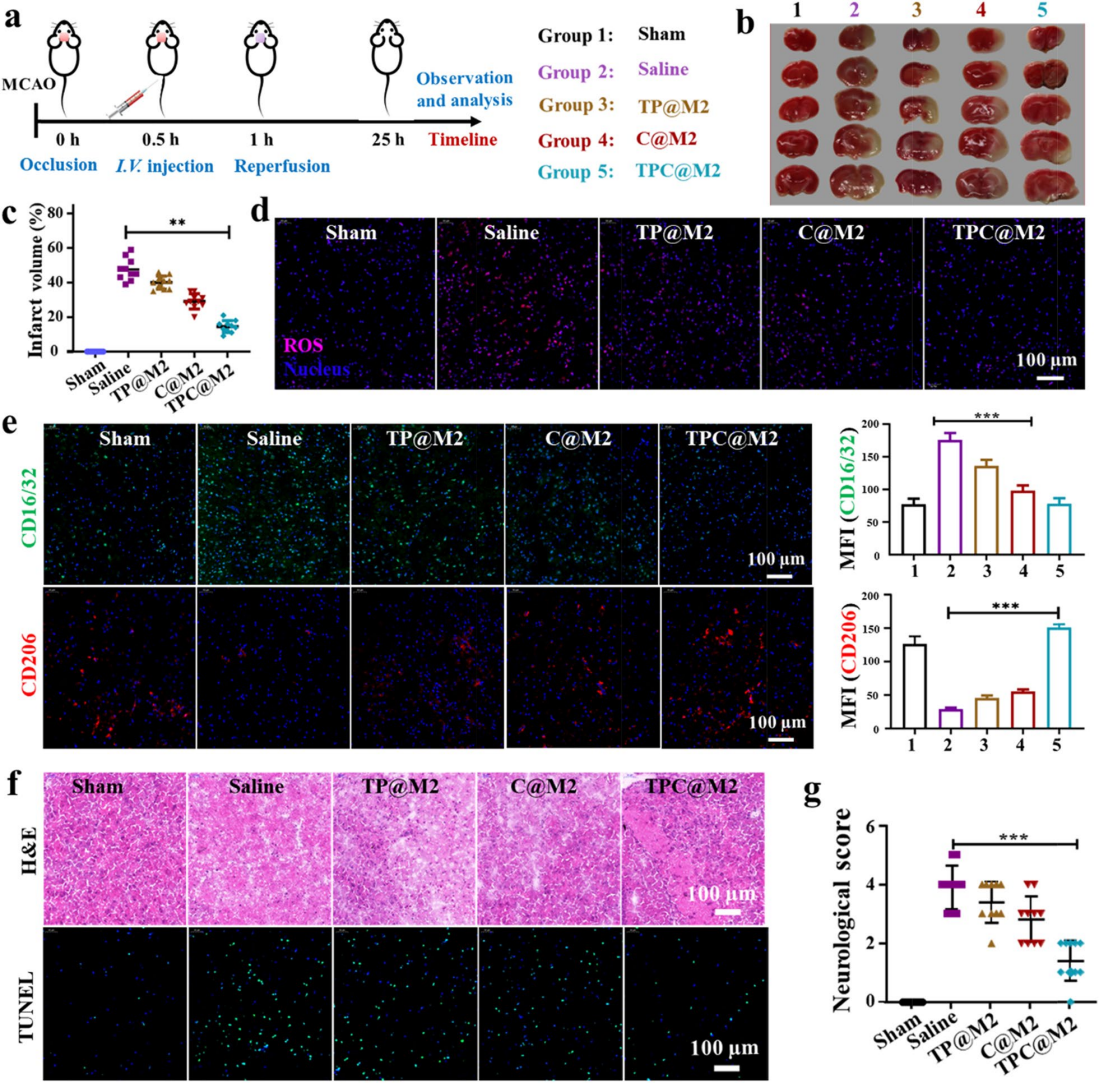
The in vivo therapeutic effects of the TPC@M2 NPs against ischemic stroke. **a** Diagram process of MCAO animal experiment. **b** Representative images of TTC-stained brain slice and **c** the corresponding quantification of infarct volume in different groups (n = 10). **d** Representative images of ROS levels in the brain after different treatments. Scale bar: 100 μm. **e** Representative images and MFI of the CD16/32 and the CD206 (red: CD206; green: CD16/32; blue: DAPI; scale bar: 100 µm) in brain treated with different groups. **f** Representative immunohistochemical analysis of the brain tissues in MCAO rats of different groups using H&E (up) and TUNEL (down) staining. **g** The neurological score of the MCAO-rat in different groups (n = 10). Results are presented as means ± s.d. *P < 0.05, **P < 0.01, and ***P < 0.001 determined by Student’s t-test

Encouraged by the above promising results, we then investigated the protective effects of the TPC@M2 NPs on stroke in vivo. According to the experimental procedure shown in Fig. 5a, MACO mice were administrated with different groups (Group 1: Sham; Group 2: Saline; Group 3: the TP@M2 NPs; Group 4: the C@M2 NPs; Group 5: the TPC@M2 NPs). After different treatments, the brains were extracted and stained with 2, 3,-5-tri phenyl tetrazole (TTC). Compared with the saline group, the TPC@M2 injected mice had a smaller cerebral infarction size (Fig. 5b). In addition, the cerebral infarct volume of the TPC@M2 NPs treated group also decreased from 50 to 15% (Fig. 5c). These results suggested that the as-fabricated TPC@M2 can protect nerve cells against ischemic stroke. Next, iron content in brain tissue was determined by graphite furnace atomic absorption spectrophotometry to investigate the scavenging effect of free iron in vivo. As shown in Additional file 1: Fig. S11, increased iron levels were observed in ipsilateral brain of MACO mice. After administration, brain iron concentration could be reduced to varying degrees in different groups, among which TPC@M2 NPs treatment group significantly reduced iron deposition. Consistent with the results, we observed a higher multiple ROS scavenging property of the TPC@M2 NPs than other groups in brains (Fig. 5d), which induced a significant increase of M2-type microglia (CD206^+^) while the proportion of M1-type microglia (CD16/32^+^) was decreased in vivo (Fig. 5e). Meanwhile, immunohistochemical analysis of the brain tissues in MCAO rats of different groups by using H&E (hematoxylin–eosin) staining and TUNEL (TdT-mediated dUTP Nick-End Labeling) assay was also conducted to confirm the therapeutic effects, where the TPC@M2 NPs showed the best anti-apoptosis and brain cell protection effect (Fig. 5f), indicating the as-designed TPC@M2 NPs holds great potential for the treatment of stroke with reperfusion injury.

More attractively, neurological scores were also detected according to Longa scoring criteria as follows: After 24 h of reperfusion, neurological function was evaluated by two observers who were blinded to the experimental procedures according to Zea-Longa scoring criteria, as follows: Score 0, no neurological injury symptoms; Score 1, contralateral forepaws of the rats cannot stretch freely (mild neurological deficits); Score 2, show contralateral rotation during walking (moderate neurological deficit); Score 3, show contralateral falling during walking (severe neurological deficits); and Score 4, fail to walk spontaneously and show loss of consciousness [21]. Compared with the saline group (4 points), the neurological score in the TPC@M2 group decreased significantly (1point, Fig. 5g), suggesting that the TPC@M2 treatment showed a significant neuroprotective effect. Finally, we investigated the in vivo biosafety of the as-fabricated NPs on the MCAO rats. The results of blood routine and serum enzyme analysis after different treatments showed no noticeable abnormalities of the TPC@M2 NPs treated rats, indicating the good biological safety of the TPC@M2 NPs (Additional file 1: Fig. S12).

## Conclusion

In conclusion, we constructed a smart but safe nano-scavenger (TPC@M2 NPs) for the highly efficient treatment of ischemic stroke. The self-assembled TPC NPs core exhibited acid-responsive properties for on-demand release of the loaded TA and the encapsulated CAT enzyme. The surface-camouflaged M2-type microglial cell membranes endowed the TPC@M2 NPs with intrinsic ischemia-homing and BBB-crossing capabilities. More importantly, the superior anti-inflammatory and neuroprotective effects of the nano-scavenger against ischemic stroke could be attributed to multiple therapeutic mechanisms which involving in scavenging over-produced multiple ROS (·OH, ·O_2_^−^, and H_2_O_2_), in situ chelating excessive Fe^2+^, and reprogramming microglia phenotype. According to our study, the as-designed TPC@M2 NPs not only had a significant inhibitory effect on the ROS-induced pro-inflammatory microglia cells but also showed an enhanced brain-targeting behavior as well as reduced ischemic brain injury in the MCAO rat model. However, the relevant research is limited, safety issues such as the biocompatibility and biodegradation of TPC@M2 NPs still need further investigate. In addition, the bioengineered nano-scavenger with multi-mechanism synergy offers a new paradigm for the management of ischemic stroke, which also reflects significant advancement in developing bioinspired nanomedicine with desirable therapeutic performances and low sides effects for the treatment of other inflammation- or CNS-related diseases. The elegant design, simple preparation process and encouraging results of the proposed nanosystem in the present study provide an effective therapeutic strategy that has potential, broad, and clinical applications for ischemic stroke.

## Experimental section

### Synthesis of the TP NPs, the TPC NPs, and the TPC@M2 NPs

The TP NPs were synthesized by self-assembly of TA and Pluronic F-68. The specific steps were as follows: Pluronic F-68 (20.00 mg) dissolved in 1 mL dimethylformamide (DMF), and its final concentration was 20.00 mg mL^−1^. TA was accurately weighed 20.00 mg and dissolved in 1 mL DMF to achieve a final concentration of 20.00 mg mL^−1^. For immediate use, Pluronic F-68 solution of 200 μL and TA solution of 200 μL were accurately absorbed into the same centrifuge tube and thoroughly mixed. Then, 40 mL of deionized water was added to a 250 mL round-bottomed flask and the mixture was added drop by drop under intense magnetic stirring at room temperature. The reaction products were immediately placed in a dialysis bag (MW120-14000) after the addition of the drip, and dialysis was conducted for 7 days with deionized water as the medium to remove free TA and DMF in the reaction, and then TP NPs nanoparticles were obtained. TPC NPs were synthesized and obtained according to the above steps by replacing the double-distilled water with double-distilled water containing CAT.

The TPC@M2 NPs were synthesized in two steps: (1) an appropriate amount of low-osmotic lysate was added to the precipitation of M2-microglia cells. The cells were homogenized 20 times with a glass homogenizer after lysing at 4 ℃ for 2 h and then centrifuged at 4 ℃ for 5 min at 3200*g*. The cell membrane in the supernatant was obtained after repeated lysis and homogenization twice. (2) The M2-microglial cell membrane was thoroughly mixed with TPC NPs, ultrasound for 3 min to obtain TPC@M2 NPs.

### Cell line and cell culture

BV-2, PC12, bEnd.3 were obtained from iCell Bioscience Inc. (Shanghai, China), they were both cultured in RPMI 1640 medium supplemented with 10% fatal bovine serum (FBS) and 1% penicillin–streptomycin solution at 37 ℃ under 5% Carbon dioxide. M2 microglia were selected from KM mice within 24 h of birth, and mixed glial cell culture was performed after extraction of primary cortical cells by purely physical methods, supplemented with nutrient stripping method. Primary mixed glial cells were cultured for 10–14 days, and after sufficient stratified cell growth was observed, microglia were isolated and purified by digestion with normal trypsin and low concentration trypsin, respectively. And then, naive microglia were collected and then stimulated with 20 ng mL^−1^ IL-4 (MCE) plus 20 ng mL^−1^ IL-13 (MCE) for 48 h to generate the M2-microglia cells.

### SDS-PAGE electrophoresis analysis protein of microglia, microglia membrane, and TPC@M2 NPs

M2 microglia cells, cell membrane, and TPC@M2 NPs were dissolved in cell lysis buffer at 4 ℃ for 30 min. The protein content was determined by the Bradford method. Sodium dodecyl sulfate–polyacrylamide gel electrophoresis (SDS-PAGE) electrophoresis analysis was performed using standard methods. Western blot analysis was conducted using the standard method.

### Confocal laser scanning microscopy images of the TPC@M2 NPs

TPC NPs labeled with FITC and microglial cell membrane labeled with Dil were thoroughly mixed with TPC NPs, ultrasound for 3 min to obtain TPC@M2 NPs, and then TPC@M2 NPs was prepared and observed under CLSM.

### Fe^2+^ coordination effect of the TP NPS

One mL of TP NP_S_ solution (1 μM, calculated by the amount of TA) was mixed with an iron standard solution of different concentrations to make the molar ratio of TP NP_S_ to Fe^2+^ from 1:1 to 1:10, and the reaction was carried out overnight at 37 ℃. The reaction products were transferred into a dialysis bag (MW1000) and dialyzed for 24 h with 20 mL deionized water as a dialysis medium. 10 mL of dialysate was taken into a 50 mL volumetric flask, and 1 mL hydroxylamine hydrochloride (10%), 2 mL 1,10-Phenanthroline (0.15%) and 5 mL NaAc solution (1 M) were added. The dialysate was diluted with deionized water to the scale, shaken well, and placed for 20 min to detect the absorbance of each sample at 510 nm wavelength. The amount of free Fe^2+^ at different molar ratios of TPC NPS to Fe^2+^ can be determined by taking the above standard curve, and then the amount of Fe^2+^ that can be complexed by 1 μM of TPC NPs at different molar ratios can be calculated by difference method.

### Catalase encapsulation efficiency

To examine the enzyme-encapsulation efficiency and loading capability, the amount of encapsulated catalase was determined by the bicinchoninic acid (BCA) Protein Assay Kit (Thermo Scientific). The BCA Protein Assay is a detergent-compatible formulation based on bicinchoninic acid (BCA) for the colorimetric detection and quantitation of total protein. The protein concentrations are determined by the absorbance at 562 nm and referring to the standards of bovine serum albumin (BSA). The concentrations of Ta were measured via inductively-coupled plasma atomic-emission spectroscopy (ICP-AES, Thermo). Catalase-encapsulation efficiency and loading capability were defined as follows:

$${\text{Encapsulation efficiency }}\left( \% \right) \, = {\text{ Mass of catalase in TPC}}@{\text{M2}}/{\text{initial mass of catalase}}.$$

$${\text{Loading capability }} = {\text{ Mass of catalase in TPC}}@{\text{M2}}/{\text{mass of TPC}}@{\text{M2}}.$$

### Release of CAT from the TPC@M2 NPs

TPC@M2 NPs (1 mg mL^−1^) incubated in pH 7.4, pH 6.5, pH 5.0 of PBS for 12 h, respectively. Centrifuged at 10,000*g* for 20 min, the supernatant was collected, and CAT content was detected according to the standard operating procedure of the catalase detection kit.

### Extracellular real-time O_2_ concentration measurement

Various concentrations of TPC@M2 NPs were added to H_2_O_2_ solutions (750 μM). The O_2_ concentration of the solution was monitored by a portable dissolved oxygen meter in real-time.

### Catalase activity assay

Catalase activity was determined by the Góth method. Briefly, 1 mL of hydrogen peroxide (50 mM) was catalyzed by adding 0.2 mL of free catalase or TPC@M2 NPs (equivalent catalase) for 1 min at 37 ℃, and then terminated by adding 1 mL of ammonium molybdate (32.4 mM) and cooling down to 25 ℃. The sample was diluted 5 times for absorbance measurement. Since ammonium molybdate reacts with the residual H_2_O_2_ to form primrose stable complexes, the relative catalase activity can be determined by the absorbance of this complex at 400 nm. For catalase activity assay, after measurement of the absorbance, the catalase activity was calculated by the following equation:

$$\begin{aligned}& {\text{The relative enzymatic activity }}\left( \% \right) \, = \, [{\text{absorbance }}({\text{1 mL H}}_{{2}} {\text{O}}_{{2}} + \, 0.{\text{2 mL PBS }} + {\text{ 1 mL ammonium molybdate}}) \, \hfill \\ &\quad - {\text{ absorbance }}\left( {{\text{1 mL H}}_{{2}} {\text{O}}_{{2}} + \, 0.{\text{2 mL TPC}}@{\text{M2 }} + {\text{ 1 mL ammonium molybdate}}} \right)\left] / \right[{\text{absorbance }}\left( {{\text{1 mL H}}_{{2}} {\text{O}}_{{2}} + \, 0.{\text{2 mL PBS }} + {\text{ 1 mL ammonium molybdate}}} \right) \hfill \\& \, \;\;\;{-}{\text{ absorbance }}\left( {{\text{1 mL H}}_{{2}} {\text{O}}_{{2}} + \, 0.{\text{2 mL free catalase }} + {\text{ 1 mL ammonium molybdate}}} \right)] \, \times { 1}00\% . \hfill \\ \end{aligned}$$

Protease K digestion for both free catalase and TPC@M2 was carried out at 37 ℃ with the final concentration of protease K at 0.4 ng mL^−1^. At predetermined time points, aliquots of the sample were removed for immediate catalase activity assay.

### H_2_O_2_ scavenging property of the TPC NPs, C@M2 NPs, and TPC@M2 NPs

TPC NPs, C@M2 NPs, and TPC@M2 NPs was dispersed in PBS containing H_2_O_2_ (10 μM) and reacted for 40 min before fluorescence measurement.

### ·O_2¯_ scavenging property of the TPC NPs, C@M2 NPs, and TPC@M2 NPs

First, xanthine (0.6 mM) and xanthine oxidase (0.05 U mL^−1^) generated ·O_2 ¯_ in phosphate buffer (0.1 M, pH 7.4) at 37 ℃ for 40 min. Then TPC NPs, C@M2 NPs, and TPC@M2 NPs were dispersed in the above solution for 40 min, then HE (0.5 ng mL^−1^) was added. Finally, the solution was thoroughly vortexed and allowed to stand for 40 min for fluorescence measurement.

### ·OH scavenging activity of the TPC NPs, C@M2 NPs, and TPC@M2 NPs

Firstly, we used FeCl_2_ and H_2_O_2_ to prepared the ·OH via Fenton reaction. 2, 2′-azino-bis(3-ethylbenzothiazoline-6-sulfonic acid) (ABTS) and EPR were employed to detected ·OH scavenging activity of TPC NPs, C@M2 NPs, and TPC@M2 NPs.

### Cytotoxicity test of the TPC@M2 NPs on BV-2 cells

BV-2 cells were spread into 96-well plates with 5 × 10^3^ cells per well and grew overnight. TPC@M2 NPs were added at a final concentration of 5, 10, 20, 50, 100 μg mL^−1^, respectively. After incubation for 24 h, add 10 μL CCK-8 to each well for 3 h of incubation, the absorbances in 450 nm were detected. Cell activity (%) = [A (lactic acid)-A(blank)]/[A (lactic acid)-A(blank)] × 100.

### Cytotoxicity test of the TPC@M2 NPs on rat primary neuronal cells

Firstly, the rat primary neuronal cells were extracted according to the standard protocol.

Then the rat primary neuronal cells were spread into 96-well plates with 5 × 10^3^ cells per well and grew overnight. TPC@M2 NPs were added at a final concentration of 5, 10, 20, 50, 100 μg mL^−1^, respectively. After incubation for 24 h, add 10 μL CCK-8 to each well for 3 h of incubation, the absorbances in 450 nm were detected. Cell activity (%) = [A (lactic acid)-A(blank)]/[A (lactic acid)-A(blank)] × 100.

### The biodistribution of TPC@M2 NPs in BV-2 cells

BV-2 cells were spread into 6-well plates with 2 × 10^5^ cells per well and grew overnight. BV-2 cell was incubated with FITC labeled TPC@M2 NPs (50 μg mL^−1^) for 0.5, 2, and 4 h, respectively. After incubation, cells were stained with Lyso-Tracker Red (75 nM) for 15 min. Then CLSM images were acquired after staining with Hoechst 33342 for 10 min (TCS SP8, Leica, Germany).

### The uptake of the TPC@M2 NPs by BV-2 cells

BV-2 cells were spread in 6-well plates with 2 × 10^5^ cells per well and grew overnight. FITC-labeled TPC@M2 NPs were added into BV-2 cell culture medium (50 μg mL^−1^), and after incubation for 0 h, 0.5 h, 2 h, 4 h, respectively, the cells were collected for fluorescence detection by flow cytometry.

### Determination of Fe^2+^ content in BV-2 cells

BV-2 cells were inoculated in confocal culture dishes with a cell density of 2 × 10^5^ cells well^−1^. Firstly, the BV-2 cells were treated with FeCl_2_ to obtain the Fe^2+^ overloaded cell model. After that, TP@M2 NPs, C@M2 NPs, and TPC@M2 NPs (TA: 50 μg mL^−1^, CAT: 50 μg mL^−1^) were added for incubation for 12 h. After incubation, PBS was washed twice, and FerroOrange was added with a fluorescent probe (1 μM) of intracellular free Fe^2+^ ions and incubated at 37 ℃ for 30 min. The intensity of intracellular red fluorescence was observed by CLSM at the excitation wavelength of 552 nm and the emission wavelength range of 570–620 nm.

### Determination of ROS content in BV-2 cells

BV-2 cells were inoculated in 6-well plates with 2 × 10^5^ cells per well. Firstly, the BV-2 cells were treated with FeCl_2_ and H_2_O_2_ to obtain the ROS overloaded cell model. After that, TP@M2 NPs, C@M2 NPs, and TPC@M2 NPs (TA: 50 μg mL^−1^, CAT: 50 μg mL^−1^) were added 12 h of incubation. After washing twice with PBS, the cells were collected for fluorescence detection by flow cytometry.

### Characterization of microglia polarization induced by the TPC@M2 NPs

Flow cytometry analysis: M1-type BV-2 cells in 6-well plates were treated with TP@M2 NPs, C@M2 NPs, and TPC@M2 NPs (TA:50 μg mL^−1^, CAT: 50 μg mL^−1^) for 48 h. After that, the cells were collected and washed twice with PBS. Cells were then incubated with CD206/CD16/32/CD11b antibody on ice for 20 min in dark. And then fluorescence was detected by flow cytometry.

Cytokine secretion: M1-type BV-2 cells in 6-well plates were treated with TP@M2 NPs, C@M2 NPs, and TPC@M2 NPs (TA: 50 μg mL^−1^, CAT: 50 μg mL^−1^) for 48 h. Then the cell supernatant of M1-type BV-2 was collected for ELISA testing of IL-10 and IL-12p70 according to standard operating procedures.

Western blot analysis: M1-type BV-2 cells in 6-well plates were treated with TP@M2 NPs, C@M2 NPs, and TPC@M2 NPs (TA: 50 μg mL^−1^, CAT: 50 μg mL^−1^) for 48 h. After being washed with PBS, the cells were collected and washed twice with PBS. Cell precipitations were lysed for 1 h at 4 °C. The iNOS protein content was measured by Bradford assay. Western blot analysis was conducted using the standard method.

Immunofluorescence analysis of NF-κB nuclear transcription: M1-type BV-2 cells in 6-well plates were treated with TP@M2 NPs, C@M2 NPs, and TPC@M2 NPs (TA: 50 μg mL^−1^, CAT: 50 μg mL^−1^) for 48 h. Then cells were incubated with NF-κB p65 antibody and DAPI to observe the NF-κB nuclear transcription.

### The transwell assay for constructing the BBB model in vitro

The BBB model was constructed by a polycarbonate 24-well transwell membrane of 0.4 μm pore size (Hanging Cell Culture Insert, Billerica, MA, USA), in which bEnd.3 cells were seeded at a density of 5 × 10^4^ cells well^−1^ and cultured for 3 days. An epithelial volt-ohm meter (Millicell-ERS-2, Millipore, USA) was used to measure the transendothelial electrical resistance of cell monolayers, and those above 220 Ω cm^−2^ were selected for experiments. Afterward, the basolateral bottom of the transwell plate was seeded with PC-12 cells. Finally, the BBB crossing ability of TPC@M2 NPs was evaluated by CLSM in vitro.

### Protective effect of polarized BV-2 cells on nerve cell PC-12

M1-type BV-2 cells (1 × 10^5^) were spread in the upper chamber, and TP@M2 NPs, C@M2 NPs and TPC@M2 NPs (TA: 50 μg mL^−1^, CAT: 50 μg mL^−1^) was added in cell culture medium. After incubation for 6 h, a fresh medium was added for further culture for 24 h. Meanwhile, PC-12 cells (2 × 10^5^) were spread in the lower chamber. PC-12 cells were collected 24 h later and cell viability was detected by CCK-8 assay.

### Mice

Adult female Sprague–Dawley (SD) rats (250 ± 20 g) were fed at the condition of 25℃ and 55% of humidity in the Experimental Animal Center of Zhengzhou University. All animal studies were performed according to the guidelines approved by the Henan laboratory animal center. The license number of Sprague–Dawley (SD) rats is SCXK (Yu) 2017-0001. All procedures were conducted following the “Guiding Principles in the Care and Use of Animals” (China) and were approved by the Laboratory Animal Ethics Committee of Zhengzhou University.

### Biodistribution of the TPC@M2 NPs in vivo

The biodistribution of TPC@M2 NPs in MCAO rats was investigated by near-infrared (NIR) fluorescence imaging in vivo. The MCAO rats were then intravenously injected with 200 µL of TPC@M2 NPs, TPC NPs (labeled with DIR) and free DIR with the same concentration (200 μg mL^−1^) as control. The fluorescence and X-ray images were collected at the predetermined time intervals post-injection (1, 5, and 10 h) by a small animal imaging system (Xtreme, Bruker, Germany). MCAO rats were then sacrificed for fluorescence imaging of the major organs (i.e. heart, liver, spleen, lung and kidney) and brain ex-vivo.

### Reprogram microglia phenotypes for neuroprotection against ischemic stroke

MCAO rat model was constructed according to the steps reported in the literature [21]. After 0.5 h of stroke, the rats were tail vein injection of 200 µL different samples (normal saline, TP@M2 NPs, C@M2 NPs, and TPC@M2 NPs) for each rat. Another 0.5 h later, the bolt was pulled out for reperfusion. After 24 h of reperfusion, the rats were sacrificed and their brains were collected and immediately frozen and cut into five or six pieces, stained with 2,3,5-tri phenyl tetrazole, and photographed with a camera. The percentage of infarct volume was analyzed by Image-Pro Plus. The mice were used to collect their brains for frozen slices. To further confirm the ROS scavenging and phenotypic transformation of microglial cells by TPC@M2 NPs in vivo, reactive oxygen species determination kit (based on DCFH-DA), CD206 and CD16/32 antibody was used to label ischemic brain regions, and DAPI was used to label the nucleus. H&E and tunnel staining were detected for histological analysis. The neurological score was detected according to Longa scoring criteria as follows: After 24 h of reperfusion, neurological function was evaluated by two observers who were blinded to the experimental procedures according to Zea-Longa scoring criteria, as follows: 0, no neurological injury symptoms; 1) contralateral forepaws of the rats cannot stretch freely (mild neurological deficits); 2) show contralateral rotation during walking (moderate neurological deficit); 3) show contralateral falling during walking (severe neurological deficits); and 4, fail to walk spontaneously and show loss of consciousness.

### Fe^2+^ scavenging potency in vivo

Total brain iron concentration (μg/g of tissue; wet weight) was measured by graphite furnace atomic absorption spectrophotometer (Z-2700, Hitachi, Japan) according to the standard protocol. Brain tissue removed 24 h after administration was cut, divided into ipsilateral and contralateral sides, and weighed. 100 mg tissue around hematoma was wet-digested with 1 mL of 1 mol/L nitric acid.

## Supplementary Information

**Additional file 1: Figure S1.** Transmission electron microscopy (TEM) image of the TP NPs at pH 7.4. Scale bar, 100 nm. **Figure S2.** Biological transmission electron microscopy (Bio-TEM) image of the TPC@M2 NPs at pH 7.4. Scale bar, 200 nm. **Figure S3.** The results of dynamic light scattering (DLS) of the TPC@M2 NPs after incubation at 37 ℃ in PBS for different times (n = 3). Results are presented as means ± s.d. *P < 0.05, **P < 0.01, and ***P < 0.001 determined by Student’s t test. **Figure S4.** The results of hemolytic experiment of the TPC NPs and TPC@M2 NPs after incubation at 37 ℃ in red cell suspension for 12 h. **Figure S4.** The results of hemolytic experiment of the TPC NPs and TPC@M2 NPs after incubation at 37℃ in red cell suspension for 12 h. **Figure S5.** The O_2_ concentration changes in H_2_O_2_ solutions (750 μM) after incubation of various concentrations of TPC@M2 NPs. **Figure S6.** The relative enzymatic activity changes of free catalase and TPC@M2 NPs after protease K digestion for different periods. **Figure S7.** Mean fluorescence intensity (MFI) of Fe^2+^ levels in BV-2 cells after different treatment (n = 3). Results are presented as means ± s.d. *P < 0.05, **P < 0.01, and ***P < 0.001 determined by Student’s t test. **Figure S8.** Representative flow cytometric analysis of the M2 biomarkers (CD206^+^) and (b) the M1 biomarkers (CD16/32^+^) on the BV-2 cells treated with different groups (n = 3). **Figure S9.** The cell viabilities of rat primary neuronal cells and PC-12 cells treated with different concentrations of the TPC@M2 NPs for 24 h (n = 6). **Figure S10.** Mean fluorescence intensity (MFI) of PC-12 cells uptaked with different treatments (n = 3). Results are presented as means ± s.d. *P < 0.05, **P < 0.01, and ***P < 0.001 determined by Student’s t test. **Figure S11.** Brain iron content at days 3 of MCAO rats in different treatment group (n = 5). Results are presented as means ± s.d. *P < 0.05, **P < 0.01, and ***P < 0.001 determined by Student’s t test. **Figure S12.** Major liver function indexes (A), kidney function indexes (B) and blood routine indexes (C) of MCAO rats in different treatment group (n = 5). Results are presented as means ± s.d. *P < 0.05, **P < 0.01, and ***P < 0.001 determined by Student’s t test.

## Data Availability

The data supporting the findings of this study are available from the corresponding author upon reasonable request.

## Abbreviations

BBB: Blood–brain barrier
BCA: Bicinchoninic acid
BSA: Bovine serum albumin
CAT: Catalase
CNS: The central nervous system
CLSM: Confocal laser scanning microscopy
DMF: Dimethylformamide
DLS: Dynamic light scattering
FBS: Fetal bovine serum
Fe^2+^: Ferrous ions
H2O2: Hydrogen peroxide
MCAO: Middle cerebral artery occlusion
NF-κB: Nuclear factor-κB
NIR: Near-infrared
·O_2¯_: Superoxide anion
·OH: Hydroxyl radical
PD: Pharmacodynamics
Pluronic F-68: Poloxamer 188
PK: Pharmacokinetics
ROS: Reactive oxygen species
rtPA: Recombinant tissue plasminogen activator
SDS-PAGE: Sodium dodecyl sulfate–polyacrylamide gel electrophoresis
TA: Tannic acid
TEM: Transmission electron microscopy
TTC: 2,3,-5-Tri phenyl tetrazole
