# Supplementary material for: An ischemia-homing bioengineered nano-scavenger for specifically alleviating multiple pathogeneses in ischemic stroke
Source: J Nanobiotechnology. 2022 Aug 31;20:397. doi: 10.1186/s12951-022-01602-7 (PMC9429703; doi:10.1186/s12951-022-01602-7)
Supplement: Supplementary file 1 — Additional file 1: Figure S1. Transmission electron microscopy (TEM) image of the TP NPs at pH 7.4. Scale bar, 100 nm. Figure S2. Biological transmission electron microscopy (Bio-TEM) image of the TPC@M2 NPs at pH 7.4. Scale bar, 200 nm. Figure S3. The results of dynamic light scattering (DLS) of the TPC@M2 NPs after incubation at 37 ℃ in PBS for different times (n = 3). Results are presented as means ± s.d. *P < 0.05, **P < 0.01, and ***P < 0.001 determined by Student’s t test. Figure S4. The results of hemolytic experiment of the TPC NPs and TPC@M2 NPs after incubation at 37 ℃ in red cell suspension for 12 h. Figure S4. The results of hemolytic experiment of the TPC NPs and TPC@M2 NPs after incubation at 37℃ in red cell suspension for 12 h. Figure S5. The O2 concentration changes in H2O2 solutions (750 μM) after incubation of various concentrations of TPC@M2 NPs. Figure S6. The relative enzymatic activity changes of free catalase and TPC@M2 NPs after protease K digestion for different periods. Figure S7. Mean fluorescence intensity (MFI) of Fe2+ levels in BV-2 cells after different treatment (n = 3). Results are presented as means ± s.d. *P < 0.05, **P < 0.01, and ***P < 0.001 determined by Student’s t test. Figure S8. Representative flow cytometric analysis of the M2 biomarkers (CD206+) and (b) the M1 biomarkers (CD16/32+) on the BV-2 cells treated with different groups (n = 3). Figure S9. The cell viabilities of rat primary neuronal cells and PC-12 cells treated with different concentrations of the TPC@M2 NPs for 24 h (n = 6). Figure S10. Mean fluorescence intensity (MFI) of PC-12 cells uptaked with different treatments (n = 3). Results are presented as means ± s.d. *P < 0.05, **P < 0.01, and ***P < 0.001 determined by Student’s t test. Figure S11. Brain iron content at days 3 of MCAO rats in different treatment group (n = 5). Results are presented as means ± s.d. *P < 0.05, **P < 0.01, and ***P < 0.001 determined by Student’s t test. Figure S12. Major liver fun [file 12951_2022_1602_MOESM1_ESM.docx]

Supporting Information

An Ischemia-Homing Bioengineered Nano-Scavenger for Specifically Alleviating Multiple Pathogeneses in Ischemic Stroke

Ranran Duan^1,†^, Ke Sun^2,^*^,†^, Fang Fang^3^, Ning Wang^4^, Ruya He^5^, Yang Gao^1^, Lijun Jing^1^, Yanfei Li^1^, Zhe Gong^1^, Yaobing Yao^1^, Tingting Luan^1^, Chaopeng Zhang^1^, Jinwei Zhang^1^, Yi Zhao^1^, Haojie Xie^1^, Yongyan Zhou^1^, Junfang Teng^1^ , Jinfeng Zhang^3,^*, Yanjie Jia^1,^*

^1^ Department of Neurology, The First Affiliated Hospital of Zhengzhou University, Zhengzhou, Henan, 450052, China

^2^ Department of Urinary surgery, The First Affiliated Hospital of Zhengzhou University, Zhengzhou, Henan, 450052, China

^3^ Key Laboratory of Molecular Medicine and Biotherapy, School of Life Sciences, Beijing Institute of Technology, Beijing, 100811, China.

^4^ Department of Biochemistry, College of Life Sciences, Shaanxi Normal University, Xi’an, Shanxi, 710062, China

^5^ The International Medical Center, The First Affiliated Hospital, Zhengzhou University, Zhengzhou, Henan, 450052, China

^†^These authors share co-first-authorship.

**Corresponding authors.**

**E-mail addresses:** [fccsunk@zzu.edu.cn](mailto:fccsunk@zzu.edu.cn) (Ke Sun), [jfzhang@bit.edu.cn](mailto:jfzhang@bit.edu.cn) (Jinfeng Zhang), [jiayanjie1971@zzu.edu.cn](mailto:jiayanjie1971@zzu.edu.cn) (Yanjie Jia)


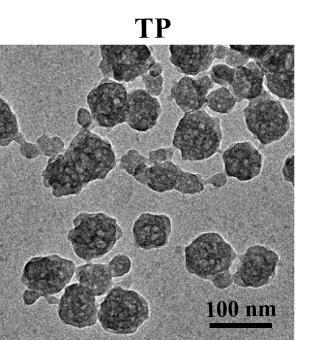


**Figure S1.** Transmission electron microscopy (TEM) image of the TP NPs at pH 7.4. Scale bar, 100 nm.


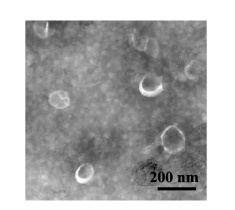


**Figure S2.** Biological transmission electron microscopy (Bio-TEM) image of the TPC@M2 NPs at pH 7.4. Scale bar, 200 nm.


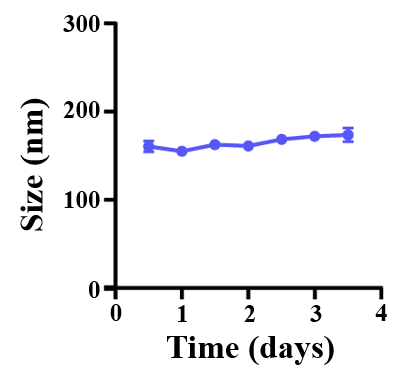


**Figure S3.** The results of dynamic light scattering (DLS) of the TPC@M2 NPs after incubation at 37℃ in PBS for different times (n=3). Results are presented as means ± s.d. *P < 0.05, **P < 0.01, and ***P < 0.001 determined by Student’s t test.


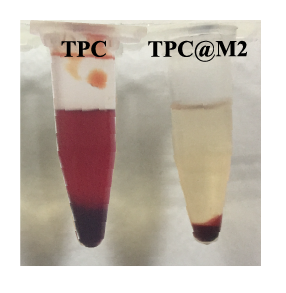


Figure S4. The results of hemolytic experiment of the TPC NPs and TPC@M2 NPs after incubation at 37℃ in red cell suspension for 12 h.


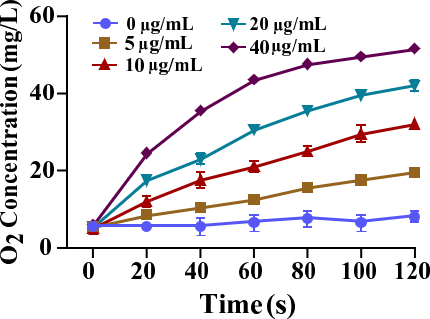


**Figure S5.** The O_2_ concentration changes in H_2_O_2_ solutions (750 μM) after incubation of various concentrations of TPC@M2 NPs.


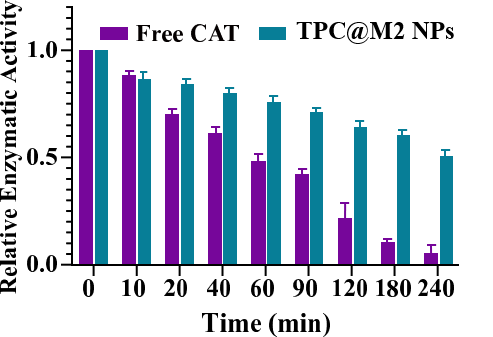


**Figure S6.** The relative enzymatic activity changes of free catalase and TPC@M2 NPs after protease K digestion for different periods.


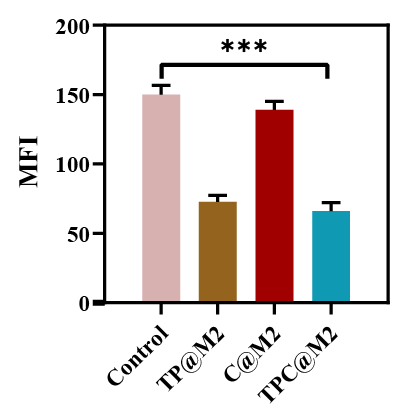


**Figure S7.** Mean fluorescence intensity (MFI) of Fe^2+^ levels in BV-2 cells after different treatment (n=3). Results are presented as means ± s.d. *P < 0.05, **P < 0.01, and ***P < 0.001 determined by Student’s t test.


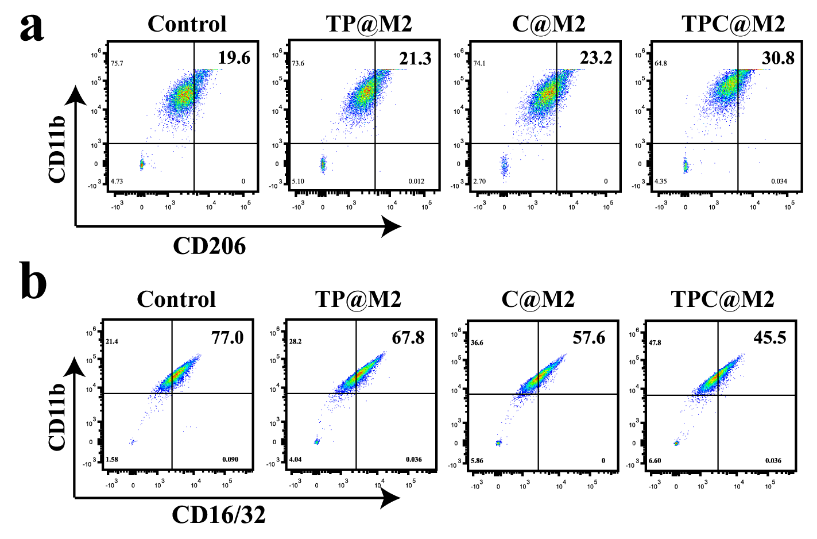


**Figure S8.** Representative flow cytometric analysis of the M2 biomarkers (CD206^+^) and (b) the M1 biomarkers (CD16/32^+^) on the BV-2 cells treated with different groups (n=3).

**Figure S9.** The cell viabilities of rat primary neuronal cells and PC-12 cells treated with different concentrations of the TPC@M2 NPs for 24 h (n=6).


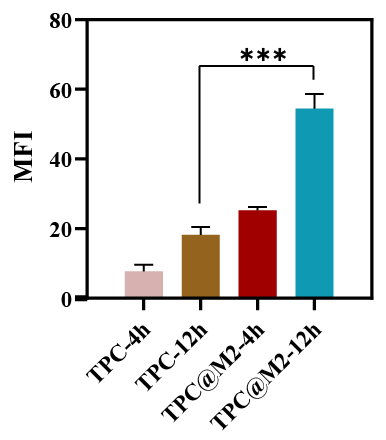


**Figure S10.** Mean fluorescence intensity (MFI) of PC-12 cells uptaked with different treatments (n=3). Results are presented as means ± s.d. *P < 0.05, **P < 0.01, and ***P < 0.001 determined by Student’s t test.

**Figure S11.** Brain iron content at days 3 of MCAO rats in different treatment group (n=5). Results are presented as means ± s.d. *P < 0.05, **P < 0.01, and ***P < 0.001 determined by Student’s t test.


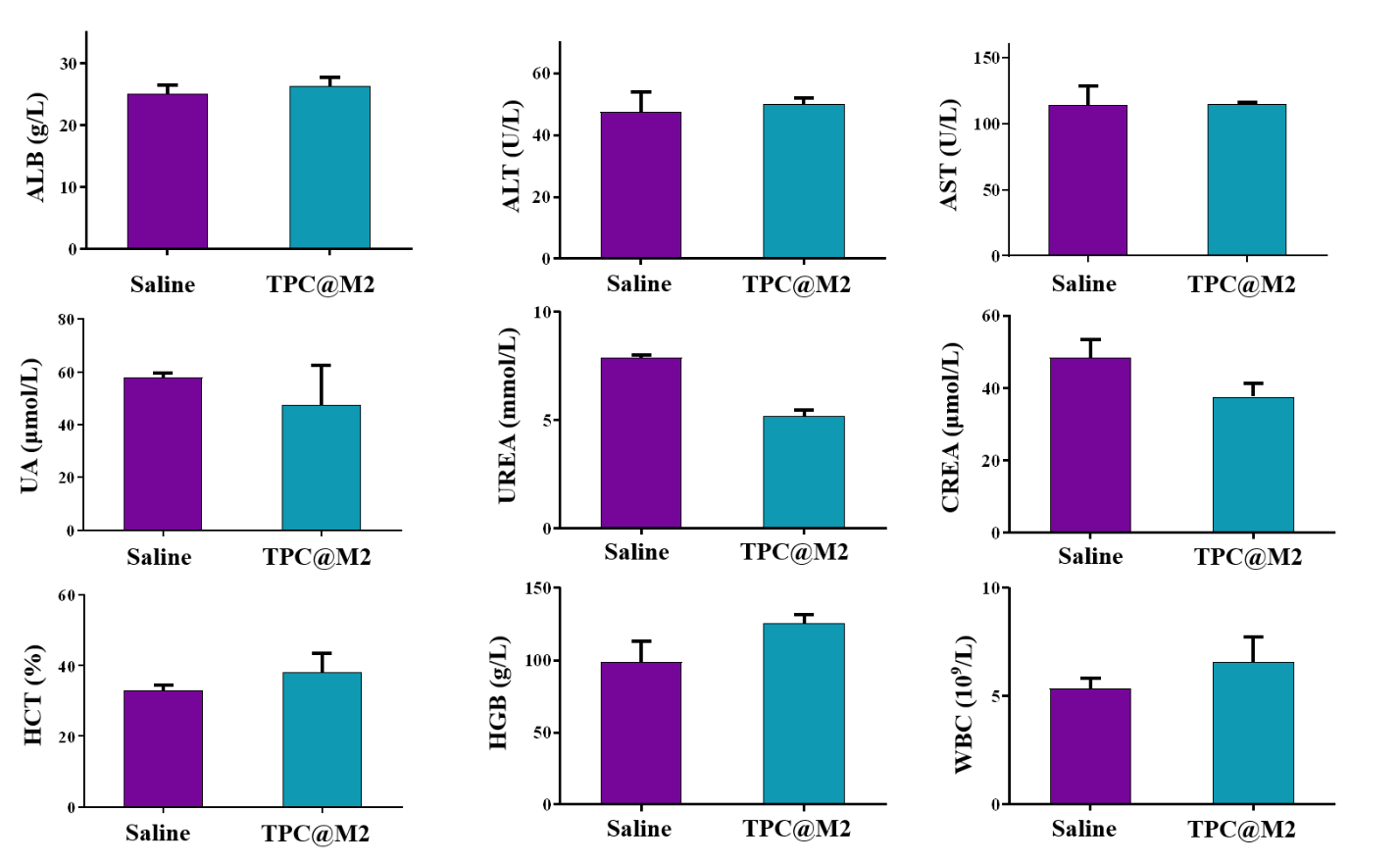


**Figure S12.** Major liver function indexes (A), kidney function indexes (B) and blood routine indexes (C) of MCAO rats in different treatment group (n=5). Results are presented as means ± s.d. *P < 0.05, **P < 0.01, and ***P < 0.001 determined by Student’s t test.
